# Hepatitis C genotype and associated risks factors of patients at University Kebangsaan Malaysia Medical Centre

**DOI:** 10.12669/pjms.295.3610

**Published:** 2013

**Authors:** N.A. Mohamed, Z Zainol Rashid, K.K. Wong, Abdullah S.A, M. M. Rahman

**Affiliations:** 1 N.A. Mohamed, Faculty of Medicine & Health Sciences, University Sains Islam Malaysia, Kuala Lumpur, Malaysia.; 2 Z. Zainol Rashid, K.K. Wong, Department of Medical Microbiology & Immunology, Faculty of Medicine, Department of Medical Microbiology & Immunology, University Kebangsaan Malaysia Medical Centre (UKMMC), Kuala Lumpur, Malaysia.; 3 K.K. Wong, Department of Medical Microbiology & Immunology, Faculty of Medicine, Department of Medical Microbiology & Immunology, University Kebangsaan Malaysia Medical Centre (UKMMC), Kuala Lumpur, Malaysia.; 4 Abdullah S.A, Department of Medicine, Department of Medical Microbiology & Immunology, Faculty of Medicine, Department of Medical Microbiology & Immunology, University Kebangsaan Malaysia Medical Centre (UKMMC), Kuala Lumpur, Malaysia.; 5 M. M. Rahman, Department of Medical Microbiology & Immunology, Faculty of Medicine, Department of Medical Microbiology & Immunology, University Kebangsaan Malaysia Medical Centre (UKMMC), Kuala Lumpur, Malaysia.

**Keywords:** RT-PCR, HCV genotype, HCV subtype, Phylogenetic analysis, Risk factors

## Abstract

***Objectives:*** Hepatitis C virus (HCV) genotyping is important for treatment and epidemiological purposes. The objective was to determine HCV genotype and their associations with certain risk factors at University Kebangsaan Malaysia Medical Centre (UKMMC).

***Methods:*** A total of 89 samples were collected from December 2009 to January 2011. Demographic data of patients were collected from medical record. Reverse Transcriptase Polymerase chain reaction (RT PCR) was performed and sixty-four samples yielded positive for HCV. Sequencing was performed and analyzed based on sequence information in GenBank. Statistical analysis were done using SPSS version 15.

***Results***: HCV genotype 3 (73%) was the most frequent genotype, followed by genotype 1(27%). The distribution of HCV genotype/ subtype was as follows: 3a (64.8%), 1a (13.5%), 1 (10.8%), 3 (8.1%) and 1b (2.7%).

***Conclusions:*** HCV subtypes 3a, 1a, and 1b were identified in patients at UKMMC, Malaysia with subtype 3a being the most prevalent. No significant association was found between HCV genotypes and patients’ demographic data.

## INTRODUCTION

World Health Organization (WHO) estimates that 150 million people worldwide are chronically infected with HCV and 3 to 4 million people are newly infected each year.^1^ In Malaysia, HCV are mostly transmitted through transfusion of contaminated blood products and intravenous drug use.^2^ A study showed that mode of transmission of HCV were associated with viral genotype.^3^ In Malaysia, report showed that HCV genotype 3(54.5%) was the most prevalent genotype followed by genotype 1 (40%). For genotype 3, 94%, 2% and 4% belonged to HCV subtype 3a, 3b and type 3, respectively. Of the HCV genotype 1, 53%, 38%, 6% and 3% belonged to subtype 1b, 1a, 1a/1b and type 1 respectively. ^4^ Studies showed that the main risk factor for infection by genotypes 1a and 3a is intravenous drug use and the main risk for genotypes 1b and 2 infections is mainly due to blood transfusion.^5^ However, there are no local data with regards to the correlation between routes of transmission and HCV genotypes.

The clinical importance of genetic heterogeneity of hepatitis C lies in the fact that certain genotypes are associated with poor response to treatment and more severe in liver pathology. Thus, HCV genotyping is recommended before starting treatment.

Treatment goals for chronic HCV infection are to delay or prevent progression of fibrosis and to prevent the development of cirrhosis. HCV genotype and virological response to treatment determine the duration of treatment. Patients with genotypes 1 and 4 are treated for 48 weeks, and those with genotypes 2 and 3 are treated for 24 weeks. Current treatment of chronic HCV infection is based on factors predicting sustained virological response (SVR).

The main aim of this study was to determine the genotype of HCV by nucleic acid sequencing and phylogenetic analysis at UKMMC. In addition associated risk factors were also assessed relating to infections of the genotypes. 

## METHODS

***Study population: ***This study was conducted with samples obtained from patients of UKMMC during December 2009 to January 2011. A total of 89 samples were sent to the laboratory of the Department of Medical Microbiology and Immunology, University Kebangsaan Malaysia for detection and characterization of hepatitis C virus. Hepatitis C virus was initially detected by Axysm® HCV version 3.0 (Abbott Technology) and finally confirmed by PCR.

***Nucleic acid extraction and PCR for HCV RNA detection: ***HCV RNA was extracted from 140 µl plasma using QIAamp® Viral RNA Mini Kit (Cat. No. 52904). The extracted samples were mixed with Qiagen One Step RT-PCR®. The primers used targeted the 5NSB region of HCV genome. The sequences of primers were: Forward primer- GCA GAA AGC GTC TAG CCA TGG CGT and reverse primer- CTC GCA AGC ACC CTA TCA GGC AGT. Then, RT-PCR was carried out in Automated Gene Amp PCR System 9700® (Applied Biosystems, USA).

***Detection of PCR product: ***A 25 µl portion of PCR product was added to 8 µl of loading dye and subsequently electrophoresed on a 2% agarose gel (SIGMA-ALDRICH, Batch # 069K1735) in TBE buffer (Promega Madison, Lot # 24224502) at 100 volt for 35 minutes. The gel was stained with GelRedTM (Lot 10G0125, Biotum, Inc). 100 bp DNA ladder (Promega Madison, G210A 21843705) and blue/orange 6 x loading dye (Promega Madison, G190A 20319009) were used to identify the specified amplified fragment. The 100base pair (bp) specific amplified bands were then visualized in ultraviolet transilluminator. 

***Sequencing: ***This was done with GenomeLab™ Dye Terminator Cycle Sequencing (Quick Start Kit) using forward primer. Procedure involved manual preparation of DNA sequencing reaction (include master mix preparation and PCR) and ethanol precipitation. Sequence analysis was done using an automated sequence analyzer CEQ™ 8000 Genetic Analysis System, Beckman Coulter.^5^

***Genotype analysis:*** The nucleotide sequence data (base-call) produced by the automated sequence analyzer CEQ™ 8000 Genetic Analysis were compared with identical sequence information in the National Center for Biotechnology Information (NCBI) based GenBank.^6^ After comparing the data in gene bank the results of subtypes were obtained.

***Phylogenetic analysis: ***The phylogenetic analysis was done by MEGA 4.0 software. Before the phylogenetic tree produced, the sequences were aligned together without group using ClustalW.

## RESULTS

A total of 89 samples were enrolled in the study and analyzed and out of which 64 samples (71%) were HCV RNA positive by RT-PCR. These 64 positive samples were subjected to sequencing and phylogenetic analysis to detect genotype. However, only 37 (68%) samples gave definite results of genotype analysis. The rest of the sequences were not adequate for analysis.

**Fig.1:**
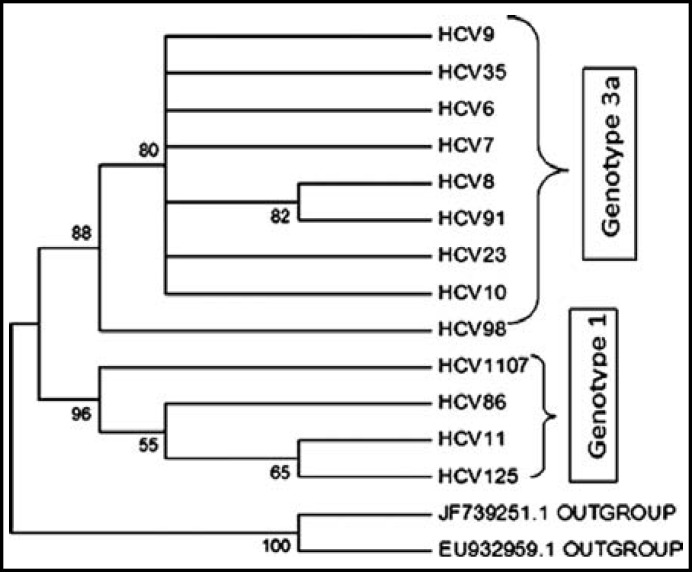
Neighbour Joining relationships of 15 taxa of HCV

**Fig.2:**
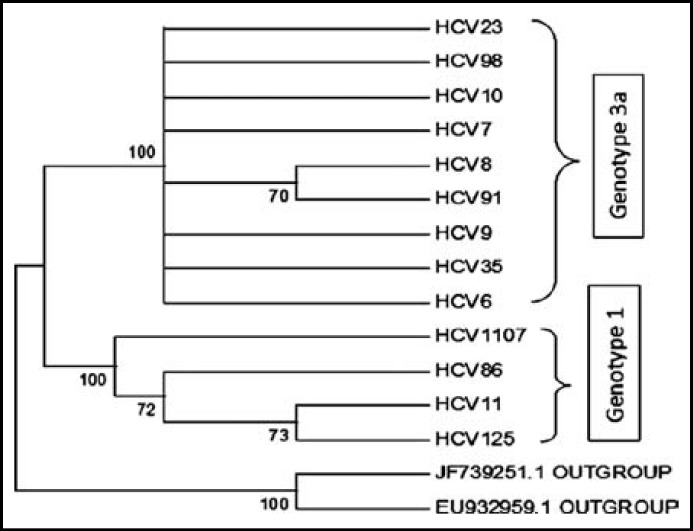
Maximum Parsimony relationships of 15 taxa of HCV

***Phylogenetic analysis: ***In 37 samples, 13 samples were accepted for phylogenetic analysis. The rest of samples were excluded due to unsatisfactory sequencing DNA data based on chromatogram results. The results are presented in Fig. 1 & 2.

Both neighbor joining and maximum parsimony phylogenetic analysis showed that all tested samples are clustered into two groups: HCV6, HCV 7, HCV 8, HCV 9, HCV 10, HCV 23, HCV 35, HCV 91 and HCV 98 are clustered in genotype 3a and HCV 11, HCV 85, HCV 125, and HCV 1107 are clustered in genotype 1.

***Distribution of HCV genotypes: ***Genotype 3 was observed in 27 out of 37 patients (73.0%) with chronic HCV infection. Of these, 24 (88.8%) patients showed infection with subtype 3a. Meanwhile, genotype 1 was determined in 10 patients (27%), 5 (13.5%) of them were infected by subtype 1a. A patient was infected with HCV subtype 1b. Seven samples could only be analyzed until genotype level; 3 (genotype 3) & 4 (genotype 1). Genotype 2, 4, 5 and 6 were not found in this study. Mean age for genotype 1 and 3 were 50.3 years and 51 years, respectively.

**Table-I:** Distribution of genotypes/subtypes

*Genotypes/Subtypes*	*Percentage (%)*
3a	65%
1a	13%
1	11%
3	8%
1b	3%

Table-I shows the distribution of 37 analyzed samples. Subtype 3a (24) was the most prevalent subtype, followed by subtype 1a (5), genotype 1 (4), genotype 3 (3) and subtype 1b (1). Associations of HCV genotypes with gender, race, mode of acquisition and co-infection were calculated using Chi-square test. No significant association was established (Table II & III).

**Table-II:** Associations of genotypes with gender and race

*Parameter*	*Genotype 1*	*Genotype 3*	*Total*	*X* ^2^	*P*
***Gender***					
Male	8(21.6)	22(59.5)	30(81.1)	0.0010	0.919
Female	2(5.4)	5(13.5)	7(19.9)		
***Race***					
Malay	4(10.8)	17(45.9)	21(56.8)	1.990	0.370
Chinese	5(13.5)	7(18.9)	12(32.4)		
Indian	1(2.7)	3(8.1)	4(10.8)		

**Table-III:** Associations of genotypes with risk factors and co-infection

***Parameter***	***Genotype 1***	***Genotype 3***	***Total***	***X *** ^2^	***P***
***Blood transfusion***					
Yes	3(8.1)	6(16.2)	9(24.3)	0.240	0.624
No	7(18.9)	21(57.0)	28(75.6)		
***Sexual promiscuity***					
Yes	1(2.7)	8(21.6)	9(24.3)	1.528	0.216
No	9(24.3)	19(51.4)	28(75.6)		
***IVDU***					
Yes	3(8.1)	7(18.90	10(27.0)	0.061	0.804
No	7(18.9)	20(54.0)	27(73.0)		
***Haemodialysis***					
Yes	1(2.7)	3(8.1)	4(10.8)	0.009	0.923
No	9(24.3)	24(64.9)	33(89.2)		
***Hepatitis B***					
Yes	0	1(2.7)	1(2.7)	0.566	0.452
No	10(20.7)	26(70.2)	36(97.2)		
***HIV***					
Yes	0	1(2.7)	1(2.70	0.566	0.452
No	10(27.0)	26(70.2)	36(97.2)		

Table-III shows the associations of HCV genotype with factors of viral acquisition that included blood transfusion, intravenous drug user (IVDU), haemodialysis and sexual promiscuity. HCV genotype in patients co-infected with hepatitis B and HIV was also demonstrated. No significant associations (p< 0.05%) was found.

## DISCUSSION

Genotype 3 (73%) was the most frequent HCV genotype in UKMMC cases. It was followed by genotype 1 which obtained from 27% of isolates. Out of the genotype 3, 89% were identified as subtype 3a. Out of the genotype 1, 60% were identified as subtype 1a. 

This result correlates with 2 studies done by the Malaysian Liver Foundation (MLF) in the year 2001 and 2003.^4^^,^^8^ HCV isolates from all over Malaysia are sent to MLF for genotyping. There is no recent data on HCV genotype in Malaysia. 

As our genotype identification results are similar to MLF that was done in 2003, therefore it could be mentioned that HCV genotype remains unchanged for the past 9 years. 

The results are also similar with studies in Thailand, India and Pakistan where majority of patients are infected with HCV genotype 3. ^9^ In Thailand, the most common subtype was 3a (39-51%), followed by subtype 1b (20-27%) and genotype 6 variants (8-18%). However a study done among blood donors showed higher prevalent of genotype 6 (31%).^10^ On the contrary, HCV 1b is the most predominant subtype in Indonesia, followed by 1c, 3k and 2a.^11^

Analysis of the genotype distribution according to age showed that patients infected with genotype 3 (mean ± SD age, 51.0 ± 10.4 years) were slightly older than those infected with genotype 1 (mean ± SD age, 50.3 ± 13.4 years). Studies showed that patients infected with genotype 1b or 2 were older than patients infected with genotype 1a, 3 and 4.^12^ Both genotype 3 (81%) and 1 (80%) were more frequent in males. Genotype analysis among major ethnic groups (Malay, Chinese and Indian) showed similar distributions; genotype 3 was predominant in all groups. Eighty one per cent (17) Malays infected with genotype 3, 11% (4) with genotype 1; 58% (7) Chinese with genotype 3, 42% (5) with genotype 1; 75% (3) Indians with genotype 3 and 25% (10) with genotype 1. Malaysian Liver Foundation data also showed that genotype 3 and 1 were found in those 3 ethnic groups, while genotype 2, 4 and 10 were detected in other minority groups.^4^

Sinniah & Ooi^13^ conducted a study among high-risk groups in Malaysia and reported that the main modes of HCV transmission identified were parenteral drug use, transfusion and/or dialysis related. Eighty five per cent from 1143 anti-HCV positive in Malaysia were IVDUs, while in 10% of cases both HCV and hepatitis B virus (HBV) were detected. Intravenous drug use (IVDU) is also the primary route of HCV transmission in the developed world. 

Risk of acquisition of HCV through blood transfusion has decreased due to screening with nucleic acid amplification method and exclusion of high-risk donor.^14^ In this study, history of blood transfusion accounted for 24% (9) of patients. Four of them received blood transfusion before 1990. HCV subtype 3a infected 44% of them, followed by genotype 1 (33%) and genotype 3 (11%). In contrast, a study done in the Netherland illustrated that subtype 1b spreads mainly by blood transfusion.^15^

HCV infection in haemodialysis population is a worrying phenomenon. Almost 10% (4) of our patients have no other risk factors apart from haemodialysis. All of them seroconverted after years of regular dialysis. Each patient was infected with different subtype/genotype; 3 (1), 3a (1), 1a (1) and 1b (1). This is comparable to a study done by Hairul^16^ in Pahang where two third of haemodialyzed patients were infected with HCV genotype 3. In contrast, recent study by Roslinda^17^ observed that the prevalence of HCV infection among haemodialyzed patients in UKM were 20% and 70% of them were infected by HCV genotype 1.

Hepatitis C virus (HCV) can be transmitted by sexual exposure but much less efficiently than other sexually transmitted viruses, including HBV and human immunodeficiency virus (HIV). Twenty four per cent of our patients gave history of sexual promiscuity. Majority of them were infected with HCV subtype 3a (78%), while genotype 1 and 3 amounted 11% each. Other risk factors include tattooing, history of operation and needle prick. Report from Mexico revealed that surgery was the primary cause for HCV infection in a tertiary hospital in Yucatan, followed by blood transfusion.^18^

This study confirmed that majority of patients with HCV infection possessed genotype 3a, followed by genotype 1a. No significant associations between risk factors and co-infection with HCV genotype were found. Genotyping based on NS5B (non-structural protein 5B) region sequence analysis could help and improve epidemiologic studies and medico-legal investigations. 

## Notes

***Disclosure of Conflicts of Interest:*** Authors declare no conflicts of interests in the study.
